# Detecting protein complexes from active protein interaction networks constructed with dynamic gene expression profiles

**DOI:** 10.1186/1477-5956-11-S1-S20

**Published:** 2013-11-07

**Authors:** Qianghua Xiao, Jianxin Wang, Xiaoqing Peng, Fang-Xiang Wu

**Affiliations:** 1 School of Information Science and Engineering, Central South University, Changsha 410083, China; 2 School of Mathematics and Physics, University of South China, HengYang 421001, China; 3 Division of Biomedical Engineering, University of Saskatchewan, Saskatoon, Canada

## Abstract

**Background:**

Protein interaction networks (PINs) are known to be useful to detect protein complexes. However, most available PINs are static, which cannot reflect the dynamic changes in real networks. At present, some researchers have tried to construct dynamic networks by incorporating time-course (dynamic) gene expression data with PINs. However, the inevitable background noise exists in the gene expression array, which could degrade the quality of dynamic networkds. Therefore, it is needed to filter out contaminated gene expression data before further data integration and analysis.

**Results:**

Firstly, we adopt a dynamic model-based method to filter noisy data from dynamic expression profiles. Then a new method is proposed for identifying active proteins from dynamic gene expression profiles. An active protein at a time point is defined as the protein the expression level of whose corresponding gene at that time point is higher than a threshold determined by a standard variance involved threshold function. Furthermore, a noise-filtered active protein interaction network (NF-APIN) is constructed. To demonstrate the efficiency of our method, we detect protein complexes from the NF-APIN, compared with those from other dynamic PINs.

**Conclusion:**

A dynamic model based method can effectively filter out noises in dynamic gene expression data. Our method to compute a threshold for determining the active time points of noise-filtered genes can make the dynamic construction more accuracy and provide a high quality framework for network analysis, such as protein complex prediction.

## Introduction

Proteomics is the most exciting frontier in life science. It becomes one of the hottest research topics in systematically analyzing and comprehensively understanding proteins through the study of protein structures, functions, and interactions [1-6]. In particular, identifying protein complexes from protein interaction networks (PINs) plays a significant role in revealing the structure of PINs, predicting protein functions, and explaining particular biological processes.

Most researches on biological networks have been focused on static networks. The static PINs, in which the interactions are accumulated in different conditions and time points, cannot reflect the real dynamic PIN networks in cell, and therefore has certain influence on the accuracy of protein complex prediction. In reality, cellular systems are highly dynamic and responsive to cues from the environments [7], and a real PIN in cell is changing over time, environments and different stages of the cell cycle[8]. In some literature [8-13], the PIN was constructed by using gene expression profiles and sub-cellular localization and other dynamic data. Time-course (dynamic) gene expression data are collected at a series of time point during a biological development process of interest and thus reflect the dynamic activity of genes during the biological development process.

In those methods[8-13], a threshold is employed to determine whether genes are significantly expressed. Nevertheless, a challenge is how to choose an appropriate threshold in order to filter out the noisy gene expression data and retain only genes which are involved in the biological development process. Tang et al. [14] have used a potential threshold to filter out noisy gene expression data. In their method the same value of a pre-defined threshold is applied to all the genes and time points. Therefore, if the minimum of a gene's expression value is greater than the threshold, the gene is judged to be always active. On the other hand, if the maximum value of a gene's expression is less than the threshold, the gene is judged to be always inactive. It is clear that this is unreasonable. Wang et al. [15] have recently introduced a 3*σ *principle to compute an active threshold for each gene based on their gene expression profiling. As a result, each gene has its own active threshold and a protein is active when its expression levels are more than its active threshold. With the notation that *µ *and *σ *are the mean and the standard deviation of a gene's expression levels, respectively, the choice of the term (*µ *+ 3*σ*) is based on the fact that the probability of the range between *±*3*σ *in a normal distribution is more than 99%. Recall that each gene has its own threshold, which is the different point from the Tang's method [14].

Although time-course gene expression data provides a dynamic snapshot of most of genes involved in a biological development process and may lead to better understanding of cellular function, not all genes on microarray are related to the biological process of interest. In addition, dynamic gene expression data is often contaminated by various noises or "noisy" genes [16]. Either excluding genes of interest or including "noisy" genes could degrade the significance of any analysis results. The challenge is how to distinguish genes of interest from a whole set of dynamic gene expression data. In this paper, we adopt a dynamic model based method to filter out noises in dynamic gene expression data. Specifically, dynamic gene expression data would be divided into two categories: one is time-dependent while another is time-independent. Time-dependent genes expression data is more likely dynamically deterministic than random while time-independent genes expression data is more likely random than dynamically deterministic. Those gene expression data are considered to be noises if they are time-independent and their means are very small.

After the contaminated genes expression data is filtered out, in this paper we use a function in the mean and the standard deviation to compute a threshold for determining the active time points of noise-filtered genes (proteins). Then we construct a noise filtered active protein interaction networks (NF-APIN) of yeast. To evaluate our method, we compare the performance of MCL on NF-APIN and TC_PIN[14] and APPIN[15]. Our proposed methods for constructing NF-APIN is described in Section Method. The computational experiments and results are presented and discussed in Section Experiments and Results. This study is concluded in Section conclusion.

## Method

In this section, we first introduce time-dependent model, time-independent model and statistic F-testing. Second, we will introduce our strategies to filter out contaminated gene expression data and deduce the active time points for each protein based on their gene expression data. Last, we construct a noise filter active protein interaction network (NF-APIN) based on the active information extracted from gene expression profile and the static PIN.

### Time-dependent model

Let *x *={*x*_1_, ..., *x_m_*, ..., *x_M_*} be a time series of observation values at equally-spaced time points from a dynamic system. AR (autoregressive) model [16] is adopted to analyze the time dependence of gene expression profiles in this paper. This study assumes that the value at time point *m *depends on the past *p *(*< m*) time points. The time-dependent relationships can be modeled by an AR model of order *p *, denoted by *AR*(*p*), which is a linear function of the values of previous *p *observations plus a term representing possible errors, i.e.,

(1) $$x_{m}=\beta_{0}+\beta_{\text{1}}x_{m-\text{1}}+\beta_{\text{2}}x_{m-\text{2}}+.\;.\;.\;\beta_{p}x_{m-p}+\varepsilon_{m};m=p+\text{1},\;.\;.\;.\;,\;M,$$

where *β_i _*(*i *= 0, 1, ..., *p*) are the autoregressive coefficients, and *ε_m _*(*m = p *+ 1, ..., *M*) represent random errors, which independently and identically follow a normal distribution with the mean of 0 and the variance of *σ*^2^. The system of Model (1) can be rewritten in the matrix form as:

(2) Y=Xβ+ε,

where $Y=\left[\begin{array}{c} x_{p+1}\\ x_{p+2}\\ \vdots \\ x_{M} \end{array}\right],X=\left[\begin{array}{cccc} 1 & x_{1} & \cdots \; & x_{p}\\ 1 & x_{2} & \cdots \; & x_{p+1}\\ 1 & \vdots & \ddots & \vdots \\ 1 & x_{M-p} & \cdots \; & x_{M-1} \end{array}\right],\beta =\left[\begin{array}{c} \beta_{0}\\ \beta_{1}\\ \vdots \\ \beta_{p} \end{array}\right],\varepsilon =\left[\begin{array}{c} \varepsilon_{p+1}\\ \varepsilon_{p+2}\\ \vdots \\ \varepsilon_{M} \end{array}\right].$

The likelihood function for Model (2) is

(3) $$L\left(\beta ,\sigma^{2}\right)={\left(2\pi \sigma^{2}\right)}^{-\left(M-p\right)/2}\text{exp}\left[-\frac{1}{2\sigma^{2}}||Y-X\beta |{|}^{2}\right].$$

If the rank (*X*) = *p *+ 1 holds, it has proved [17] that the maximum likelihood estimates of *β *and *σ*^2 ^are

(4) $$\hat{\beta }={\left(X^{T}X\right)}^{-1}X^{T}Y$$

and

(5) $${\hat{\sigma }}^{2}=\;||Y-X\hat{\beta }|{|}^{2}/\left(M-p\right).$$

The value of the maximum likelihood is given by

(6) $$L\left(\hat{\beta },{\hat{\sigma }}^{2}\right)={{\left(2\pi {\hat{\sigma }}^{2}\right)}^{-}}^{\left(M-p\right)/2}{e^{-}}^{\left(M-p\right)/2}.$$

In Model (2), the matrix *X *has *p *+ 1 columns and *M − p *rows. Thus a necessary condition for *rank*(*X*) = *p *+ 1 is *M − p ≥ p *+ 1 or *p ≤ *(*M − *1)*/*2.

### Time-independent model

For a group of observation values which are not produced by the dynamic systems under consideration, but noisy (random) data, one can simply model them by a constant number plus random errors. Let *x *= {*x*_1_, ..., *x_m_*, ..., *x_M_*} be a series of time independent (random) observations. In agreement with Model (2), the last (*M − p*) observations can be modeled by

(7) $$x_{m}=\beta_{0}+\varepsilon_{m},m=p\ldots ,M,$$

where *β*_0 _is a constant number and *ε_m_*(*m = p*, ..., *M *) are the random errors which are subject to a normal distribution independent of time with the mean of 0 and the variance of $\sigma_{c}^{2}$. The likelihood function for Model (7) is

(8) $$L\left(\beta_{0},\sigma_{c}^{2}\right)={\left(2\pi \sigma_{c}^{2}\right)}^{-\left(M-p\right)/2}\text{exp}\left[-\frac{1}{2\sigma_{c}^{2}}\overset{M}{\underset{m=p+1}{\sum }}{\left(x_{m}-\beta_{0}\right)}^{2}\right].$$

The maximum likelihood estimates of *β*_0 _and $\sigma_{c}^{2}$ are

(9) $${\hat{\beta }}_{0}=\frac{1}{M-p}\overset{M}{\underset{m=p+1}{\sum }}x_{m}$$

and

(10) $${\hat{\sigma }}_{c}^{2}=\frac{1}{\left(M-p\right)}\overset{M}{\underset{m=p+1}{\sum }}{\left(x_{m}-{\hat{\beta }}_{0}\right)}^{2}$$

respectively. The maximum values of the likelihood is given by

(11) $$L\left({\hat{\beta }}_{c},{\left({\hat{\sigma }}_{c}\right)}^{2}\right)={\left(2\pi {\left({\hat{\sigma }}_{c}\right)}^{2}\right)}^{-\left(M-p\right)/2}e^{-\left(M-p\right)/2},$$

where ${\hat{\beta }}_{c}$ is a (*p *+ 1) dimensional vector whose first component is ${\hat{\beta }}_{0}$and others are zeros.

### Hypothesis testing

Actually, the time-independent model is also an autoregressive model with the order of zero and can be viewed as Model (1) with constraints *β_i _*= 0 (*i *= 1, ..., *p*). These constraints can be rewritten in the matrix form as follows

(12) Aβ=0,

where $A=\left[\begin{array}{ccccc} 0 & 1 & 0 & \cdots \; & 0\\ 0 & 0 & 1 & \cdots \; & 0\\ \cdots \; & \cdots \; & \cdots \; & \ddots & \vdots \\ 0 & 0 & 0 & 0 & 1 \end{array}\right].$

The likelihood ratio of Model (7) to Model (1) is given by

(13) $$\Lambda =\frac{L\left({\hat{\beta }}_{c},{\left({\hat{\sigma }}_{c}\right)}^{2}\right)}{L\left(\hat{\beta },{\left(\hat{\sigma }\right)}^{2}\right)}={\left(\frac{{\left(\hat{\sigma }\right)}^{2}}{{\left({\hat{\sigma }}_{c}\right)}^{2}}\right)}^{\left(M-p\right)/2}.$$

As Model (7) can be viewed as a regression Model (1) with the Constraints (12), and the maximum likelihood method is used to obtain ${\left(\hat{\sigma }\right)}^{2}$ and ${\left({\hat{\sigma }}_{c}\right)}^{2}$, the inequality ${\left(\hat{\sigma }\right)}^{2}\leq {\left({\hat{\sigma }}_{c}\right)}^{2}$ comes true. According to the likelihood principle [18], if Λ in Formula (13) is too small, Model (1) is preferable, i.e. the series *x *= {*x*_1_, ..., *x_m_*, ..., *x_M_*} is more likely time-dependent than time-independent. Although Λ in Formula (13) is not a convenient test statistic, it has proved [17] that the statistic

(14) $$F=\frac{M-2p-1}{p}\left({\text{Λ}}^{-2/\left(M-p\right)}-1\right)=\frac{M-2p-1}{p}\left(\frac{{\hat{\sigma }}_{c}^{2}}{{\hat{\sigma }}^{2}}-1\right)$$

follows an *F *distribution with (*p, M − *2*p − *1) degrees of freedom when Model (7) is true for a series of observations. When *F *is very large, thus the *p*-value is very small (Here, the *p*-value is the probability of obtaining a test statistic at least as extreme as the one that was actually observed, assuming that the null hypothesis is true. One often "rejects the null hypothesis" when the p-value is less than the predetermined significance level), Model (7) is rejected, i.e., observation series *x *= {*x*_1_, ..., *x_m_*, ..., *x_M_*} is time-dependent. From Formula (14), one can calculate the probability (*p*-value) that a series of observations is not time-independent. As the regression degree in Model (1) is unknown, the *p*-values are calculated by Formula (14) for all possible orders *p *(1 *≤ p ≤ *(*M − *1)*/*2). The proposed method calls a gene to be significantly expressed (time-dependent) if one of these *p*-values calculated from its expression profile is smaller than a user-preset threshold value.

### The steps for constructing noise filtered active protein interaction networks

#### Filtering noisy genes

Gene expression profiles will be divided into two categories by using time-dependent model and time-independent model described in the previous subsection at the first step. It is understandable that a gene expression profile is time-dependent if it can be best modeled by a non zero-order AR equation, while a gene expression profile is time-independent if it can be best modeled by a zero-order AR equation. A gene will be considered being noise if the gene expression data belongs to time-independent and its mean is very small. Thus, the definition of "small" is very important. Our strategy is that, firstly all of genes belong to time-independent are sorted ascending by their means of genes expression data. Then, given a threshold value, a gene is considered being noise if the mean of gene expression value is less than the threshold. In this study, genes with the top 15% of the lowest mean are judged as the noisy genes, As a result, the mean threshold is set as 0.5. The reason of choice of 0.5 here will also be further discussed in effect of the coefficient *k *selection of this paper later on.

#### Filtering gene expression data point

Usually, the threshold in gene expression array is used to differentiate false-positive expressed gene (noise) from true-positive expressed gene[14-27]. Tang et al. [14] have proposed a simple threshold, which returns a constant real number for any gene at any time point. In other words, the common threshold is applied to all the genes at different time points. Although their approach works better than existing methods at that time, the common threshold might be not efficient for all the genes. For example, most proteins with low expression values will be filtered out. Wang et al. [15] have devised a threshold function based on the mean and standard deviation of expression levels of a gene. Their threshold function is defined as follows:

(15) Active_threshold=u+3σ×(1-F)

(16) $$F=\frac{1}{1+\sigma^{2}}$$

For each gene, *u *and *σ *are the mean and standard deviation of its expression values. If the fluctuation of expression values is high, corresponding to a high value of *σ *and thus small *F*, the threshold may be greater than its all expression values. In other words, some proteins with high fluctuation will be filtered out.

In this subsection, we describe a way to determine whether a protein at a time point is active from dynamic expression levels of the corresponding gene. Our threshold function is described as follows:

(17) Active_threshold=u+kσ×(1-F)

Three standard deviations include about 99% of all observations. On the other words, in normal case only less than 1% of the time point may be active. In summary, in the same case, the threshold by using parameter *k × σ *is less than by using parameter 3*σ*. More gene expressing profiles will be retained. The *Active_threshold *is calculated by Formula (17) for all possible values *k *(0 *≤ k ≤ *3). In this paper the value of coefficient *k *is selected as 2.5. The reason of why select 2.5 as the value of coefficient *k *will be discussed in the section *effect of the coefficient k selection *of this paper later on.

#### Construction of NF-APIN

Two proteins interacted in the static PIN may not interact with each other all the time in a dynamic network, because they may not always active at the same time. Dynamic network aims at reflecting dynamic interactions between proteins, which are changing with time and condition. The dynamic interactions are determined by the dynamics of protein activity. If the expression level of a gene is over its active threshold at a time point, the corresponding protein is regarded as active at the time point. For each time point, if two proteins interacted with each other in the static PPI network are active at the same time point, the proteins and their interaction form a part of NF-APIN at the time point. The process is repeated until the NF-APIN is created.

## Experiments and results

In this section, we first construct an NF-APIN. Then we compare the efficiency of three dynamic network, NF-APIN, APPIN (Wang et al., [15]) and TC-PIN (Tang et al., [14]), by applying a clustering method to identify protein complexes.

### Material

Protein interactions of many species are available, particularly in the model organism Saccharomyces cerevisiae (a strain of yeast). Since the relationship between proteins and genes of yeast is almost unique mapping, no need to consider the different combination of exons, and the genome of yeast have been well understood, the gene expression array of yeast can provide a comprehensive view of protein expression. Therefore, we construct an NF-APIN of yeast. The genome-wide set of PPIs of the yeast are downloaded from DIP [28] updated to Oct. 10, 2010. As customary, self interactions and duplicated interactions should be discarded. Therefore, the static PIN contains 5093 proteins and 24743 interactions. We use GSE3431 in gene Expression Omnibus (GEO) [29], which is a gene expression profiling of yeast over three successive metabolic cycles. The 6,777 gene products in the gene expressing profile cover 95% of the proteins in the PPI network. That is to say, 4846 gene expression profiles are used in our experiment. There are 3 cycles in this expression experiment. For each cycle there are 12 time time points, and the time interval between two time points is 25 minutes. Thus each gene has 12 gene expression values (levels) in each cycle. In our experiment, we use the gene expression of 3 cycles to filter noisy genes. Hence there are 36 time points for our AR model. Since in GSE3431, the cycles are successive, we use one cycle with average expression value of every time point of three cycles to compute *Active_threshold *and determine if a gene is active at a time point.

### Network construction

In TC-PIN [14], a potential threshold is set as 0.7, and for all the genes the gene expression levels at different time points are compared with it. A protein is considered to be expressed at a time point when the gene expression level of its corresponding gene is greater than the threshold; otherwise, the protein is considered to be unexpressed at the time point. For each time point, there is a subnetwork which is constituted by the interactions derived from the static PIN, the protein pairs of which are both expressed. Similar with TC-PIN, APPIN [15] contains subnetworks of each time point. The difference is that APPIN [15] uses an active threshold to determine the active time points for each protein according to the characteristics of its gene expression curve, rather than a global threshold. In the construction of NF-APIN, we divide gene expression profiles into two categories by using time-dependent model and time-independent model. In our experiments, the value of order *p *is up to 6 in AR model and the *p*-value is equal to 0.01 in *F*-testing. 19.4% gene expression profiles is time-dependent, 80.6% gene expression profiles is the time-independent. About 15% genes belong to time-independent are identified as noisy genes because of their small means. *Active_threshold *is calculated by Formula (17). For all possible values k (0 *≤ k ≤ *3), k is selected as 2.5 in our experiment. Since many proteins are not active at the same time point resulting a small subset of efficacious interactions at the time point, these subnetworks in NF-APIN contains 646 nodes and 1101 edges on average while those in APPIN contains 776 nodes and 1281 edges on average and in TC-PIN contains 3558 nodes and 16961 edges on average. Compared with APPIN and TC-PIN, the average numbers of nodes and edges of the subnetworks of TC-PIN are about 5.5 times and 15.4 times than those of NF-APIN, and the average numbers of nodes and edges of the subnetworks of NF-APIN are a little less than those in APPIN.

### *Effect of the coefficient *k *selection*

**Figure 1:**
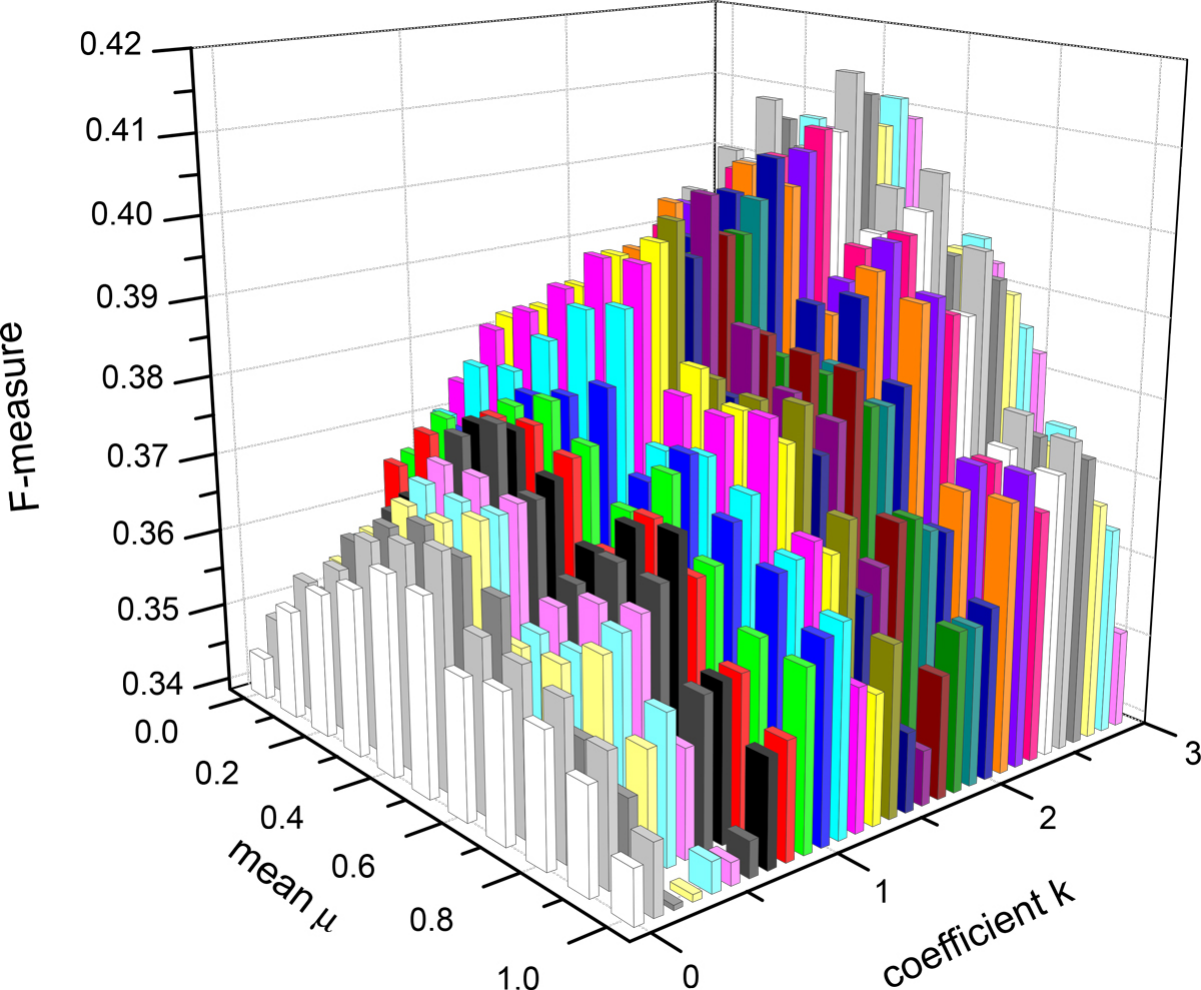
***f*-measure of MCL about *k* from 0 to 3, the interval is 0.1**. Figure 1 shows how the different values of coefficient k and mean adopting in the refinement effect f-measure. the coefficient k ranges from 0 to 3 with the increment of 0.1 and the mean ranges from 0 to 1 with the increment of 0.1 too.

In this paper, we assume unlike in [15] that a protein might be active if its corresponding gene expression values are greater than its mean, plus less than three standard deviations. Instead, we consider that the value of the coefficient *k *is greater than or equal to 0 and less than or equal to 3. To investigate the effect of values of *k*, we conduct experiments in various values of *k *and the mean. Specifically, the coefficient *k *ranges from 0 to 3 with the increment of 0.1 and the mean ranges from 0 to 1 with the increment of 0.1 too. As the coefficient *k *rises, the *Active_threshold *also rises. With the increasing number of filtered genes, the number of the new functional modules also increases. As shown in Figure 1, it can be seen that when a mean value falls within the region from 0.4 to 0.6, the value of *f*-measure of MCL is achieved to an optimal result in a fixed coefficient *k*. In other words, the number of noisy genes filtered out achieves to an optimal result. At the same time, it can be seen that when the value of coefficient *k *is in the vicinity of 2.5, the value of *f*-measure of MCL is achieved to an optimal result. Therefore, 2.5 is chosen as the coefficient *k *in our study.

### Comparison with the known complexes

The Markov Cluster algorithm (MCL) [30,31] is a clustering method by simulating a flow on the graph by calculating successive powers of the associated adjacency matrix, and is applied to extracting clusters from the graph. MCL is robust clustering algorithms[32,33], therefore it can be applied to evaluate the efficiency of different networks without bias. Wang et al. and Tang et al. have applied MCL to identify protein complexes.We also apply MCL to identify protein complexes, and compare the performances of MCL on NF-APIN, TC-PIN, and APPIN. For three dynamic networks, each of them contains 12 subnetworks. For each dynamic PIN, we first apply MCL to predict complexes on each subnetwork, then combine the prediction complexes based on a overlap strategy. There are 408 benchmark complexes considered as the gold standard data [34]. Table 1 lists the number of complexes predicted by MCL on each dynamic network, the number of known complexes matched by clusters when *OS *≥ 0.2 or *OS *≥ 1. Other metrics, such as *sensitivity, specificity *and *f*-measure, respectively when *OS *≥ 0.2 [35-37], are also compared in Table 1. It is easy to find out the *sensitivity *of MCL achieved on NF-APIN is much higher than that on TC-PIN 2. while it is slightly less than that on APPIN. Remarkably, *specificity *and *f*-measure of MCL achieved on NF-APIN are much better than those achieved on other two dynamic networks.

**Table 1:** The Performance Comparison of MCL on three dynamic networks.

Network	#*P C*	#*M KC *(*OS *= 0.2)	#*M KC *(*OS *= 1)	*Sn*	*Sp*	*F*
NF-APIN	1686	235	33	0.738	0.289	0.415
APPIN	2013	256	37	0.773	0.257	0.386
TC-PIN	2034	220	23	0.696	0.212	0.326

*OS *is the overlapping score, #*PC *is the number of clusters predicted by MCL in each network, and #*MKC *is the number of known complexes matched by clusters when *OS *≥ 0.2 or *OS *≥ 1. The sensitivity, specificity and *f*-measure are listed in the table when *OS *≥ 0.2.

### Go function enrichment analysis

The GO function enrichment analysis is used to evaluate the functional relevance of the clusters predicted from PINs with a *p − value*. In our experiments, the *p − value *[37-39] of clusters are calculated by a tool, GO Term Finder (http://www.yeastgenome.org). A predicted protein complex is considered to be significant if its P-value *≤ *0.01. To evaluate the efficiency of different dynamic networks, we analyze the function enrichment of protein complexes predicted from three networks. Since the predicted protein complex whose *p − value *is more than 0.01 are considered to have little biological significance, in TABLE 2, the ratios of significant protein complexes with *p − value *are calculated in five different intervals: *<*E-15), [E-15, E-10), [E-10, E-5), [E-5, 0.01) and *≤*0.01. Table 2 shows that the total ratio of significant protein complexes predicted from NF-APIN is up to 42% in aspect of *B.P*., which is higher than APPIN and TC-PIN. It demonstrates that the protein complexes predicted from NF-APIN are more enriched with the functions of biological process, which also indicates the better quality of NF-APIN.

**Table 2:** Functional Enrichment (B.P.) of the Identified Clusters.

	*<*E-15	E-15 to E-10	E-10 to E-5	E-5 to 0.01	*≥*0.01
NF-APIN	0.4%	1.5%	14.3%	25.4%	58.4%
APPIN	0.3%	1.1%	8.2%	28.3%	61.9%
TC-PIN	2.3%	3.5%	11.9%	23.5%	58.8%

We analyze the enrichment of biological function of clusters predicted on APPIN and TC-PIN and NF-APIN. Table 2 shows the percentage of the significant clusters from three dynamic networks in aspect B.P.

### Comparison in accuracy

For each predicted protein complex, the *precision, recall *and *F − score *are calculated for the function it be assigned. *F − score *is defined as the ratio of the square of the geometric average of precision (*p*)[40] and recall (*r*) to their arithmetic average, which is used to evaluate the accuracy of the complex identified from PIN. The Gene Ontology contains three domains: Biological Process (*B.P*.), Cellular Component (*C.C*) and Molecular Function (*M.F*.). In this subsection, we compare the average accuracies of predicted protein complexes on *B.P*., *M.F*. and *C.C *on each dynamic network. As is shown in TABLE 3, the accuracy of each domain of protein complexes predicted on NF-APIN is better than that on APPIN and TC-PIN.

**Table 3:** Comparison of the Accuracies of MCL.

	Accuracy		
	**B.P**.	**M.F**.	**C.C**.
NF-APIN	0.52	0.30	0.62
APPIN	0.49	0.27	0.59
TC-PIN	0.24	0.18	0.36

The recall and precision is used to evaluate the accuracy of the identified complexes in complex detection. We compare the accuracies of MCL on B.P., M.F., and C.C.

## Conclusion

This paper first presents a dynamic model-based approach to distinguish time-dependent gene expression data from time-independent gene expression data. In the proposed method, a time-course gene expression profile is described by an autoregressive model. The order of autoregressive model, the mean of genes expression data and *k *may changes with different genes, therefore all possible order, reasonable mean and reasonable *k *of the autoregressive models are tested in this work. Based on these results, we further devise a method for selecting active proteins at each time point, in which the active threshold for each protein is calculated by a function with the mean and the standard deviation of their corresponding gene expression data. Finally a NF-APIN is constructed by incorporating static PINs with selected active proteins, which is consisted by a number of subnetworks. To investigate the quality of NF-APIN, we apply a clustering method on NF-APIN to detect the protein complexes, and compare it with its competing dynamic networks, APPIN and TC-PIN. The results in TABLE 1-3 illustrates that NF-APIN is a more precise and biological network for detecting protein complexes than other dynamic networks. The superior performance on NF-APIN can attribute to the accurate active protein information and efficacious interactions based on our presented dynamic model-based approach.

Besides complex prediction, NF-APIN can be also applied for other network analysis, such as pathway inference, essential protein, protein function, and disease diagnose and prediction. With more and more PINs and gene expression data under special environments of other species available, we can construct NF-APINs for different species with different research purposes. With more and more dynamic information, we can construct NF-APINs for different research purposes. In addition, we also need more direct information about proteins in biological experiments to validate and support our method and to improve accuracy of filtering noisy data. In further study, we will employ this strategy to investigate protein networks of other species and gene expression arrays under special environments.

## Competing interests

The authors declare that they have no competing interests.

## Authors' contributions

QX and JW obtained the protein-protein interaction data and gene expression data, generated the prediction model and drafted the manuscript. QX and XP performed experimental comparison and evaluated the results. JW, QX, FW initiate the study and write the manuscript. All authors have read and approved the final manuscript.

## Declarations section

The publication costs for this article were funded by the corresponding author

This article has been published as part of *Proteome Science *Volume 11 Supplement 1, 2013: Selected articles from the IEEE International Conference on Bioinformatics and Biomedicine 2012: Proteome Science. The full contents of the supplement are available online at http://www.proteomesci.com/supplements/11/S1.
